# *N*-Benzyl-1,3-dide­oxy-1,3-imino-l-xylitol

**DOI:** 10.1107/S160053681103399X

**Published:** 2011-08-27

**Authors:** Sarah F. Jenkinson, Gabriel M. J. Lenagh-Snow, George W. J. Fleet, Amber L. Thompson

**Affiliations:** a Department of Chemistry, Chemistry Research Laboratory, University of Oxford, Oxford OX1 3TA, England; b Department of Chemical Crystallography, Chemistry Research Laboratory, University of Oxford, Oxford OX1 3TA, England

## Abstract

The structure determination confirms the stereochemistry of the title compound, C_12_H_17_NO_3_, which contains a four-membered azetidine ring system. The absolute configuration was determined by the use of d-glucose as the starting material. In the crystal, O—H⋯O and O—H⋯N hydrogen bonds link the mol­ecules into layers in the *ab* plane.

## Related literature

For related literature on azetidines, see: Krämer *et al.* (1997 ▶); Michaud *et al.* (1997*a* ▶,*b* ▶); Dekaris & Reissig (2010 ▶); Soengas *et al.* (2011 ▶). For related literature on imino­sugars, see: Asano *et al.* (2000 ▶); Watson *et al.* (2001 ▶). For details of the cryostat, see: Cosier & Glazer (1986 ▶).
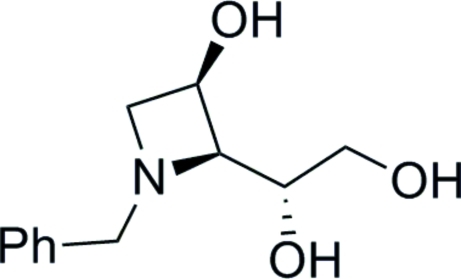


## Experimental

### 

#### Crystal data

C_12_H_17_NO_3_
*M* *_r_* = 223.27
Orthorhombic, 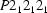

*a* = 6.2309 (2) Å
*b* = 9.3918 (4) Å
*c* = 19.9175 (9) Å
*V* = 1165.56 (8) Å^3^
*Z* = 4
Mo *K*α radiation
μ = 0.09 mm^−1^
*T* = 150 K
0.20 × 0.10 × 0.07 mm

#### Data collection

Nonius KappaCCD diffractometer
Absorption correction: multi-scan (*DENZO*/*SCALEPACK*; Otwinowski & Minor, 1997 ▶) *T*_min_ = 0.97, *T*_max_ = 0.99
6149 measured reflections
1541 independent reflections
1098 reflections with *I* > 2σ(*I*)
*R*_int_ = 0.065

#### Refinement

*R*[*F* ^2^ > 2σ(*F* ^2^)] = 0.045
*wR*(*F* ^2^) = 0.102
*S* = 0.95
1541 reflections
146 parameters
H-atom parameters constrained
Δρ_max_ = 0.49 e Å^−3^
Δρ_min_ = −0.49 e Å^−3^

### 

Data collection: *COLLECT* (Nonius, 2001 ▶); cell refinement: *DENZO*/*SCALEPACK* (Otwinowski & Minor, 1997 ▶); data reduction: *DENZO*/*SCALEPACK*; program(s) used to solve structure: *SIR92* (Altomare *et al.*, 1994 ▶); program(s) used to refine structure: *CRYSTALS* (Betteridge *et al.*, 2003 ▶); molecular graphics: *CAMERON* (Watkin *et al.*, 1996 ▶); software used to prepare material for publication: *CRYSTALS*.

## Supplementary Material

Crystal structure: contains datablock(s) global, I. DOI: 10.1107/S160053681103399X/lh5318sup1.cif

Structure factors: contains datablock(s) I. DOI: 10.1107/S160053681103399X/lh5318Isup2.hkl

Additional supplementary materials:  crystallographic information; 3D view; checkCIF report

## Figures and Tables

**Table 1:** Hydrogen-bond geometry (Å, °)

*D*—H⋯*A*	*D*—H	H⋯*A*	*D*⋯*A*	*D*—H⋯*A*
O4—H41⋯O16^i^	0.84	2.18	2.825 (4)	134
O16—H161⋯O1^ii^	0.84	1.90	2.735 (4)	171
O1—H11⋯N6^i^	0.86	1.86	2.719 (4)	171

Symmetry codes: (i) 
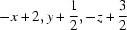
; (ii) 
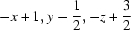
.

**Fig. 1.**
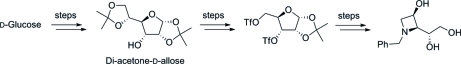
Synthetic Scheme.

**Fig. 2.**
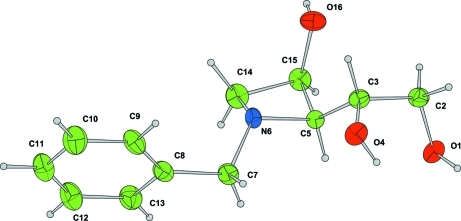
The title compound with displacement ellipsoids drawn at the 50% probability level. H atoms are shown as spheres of arbitary radius.

**Fig. 3.**
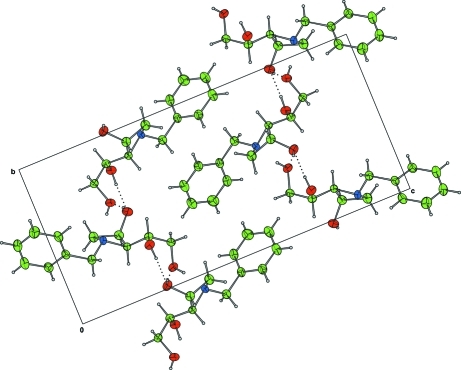
Packing diagram of the compound projected along the a axis. Hydrogen bonding is denoted by dotted lines.

**Fig. 4.**
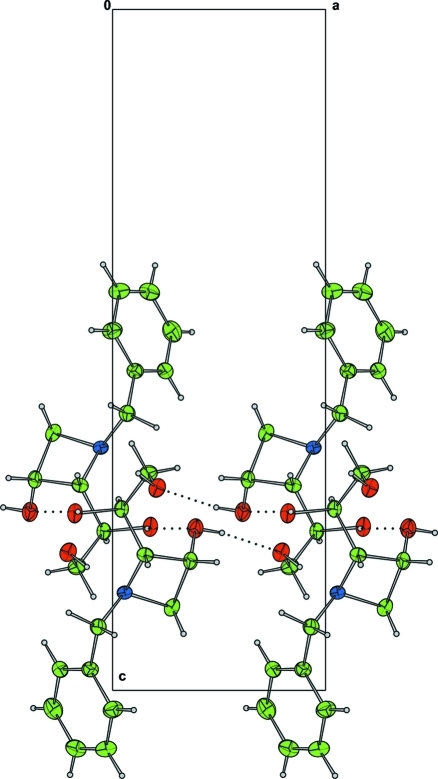
Packing diagram of the compound projected along the b axis. Hydrogen bonding is denoted by dotted lines.

## supplementary crystallographic information

### Comment

Azetidines (Michaud *et al.*, 1997*a*,*b*; Dekaris & Reissig, 2010; Soengas *et al.*, 2011) are a relatively unstudied class of iminosugars (Asano *et al.*, 2000; Watson *et al.*, 2001) but initial results (Krämer *et al.*, 1997) have shown that they can exhibit interesting biological activity. The title compound was formed from a protected ribofuranose derived from *D*-glucose (Fig. 1).

The molecular structure of the title compound is shown in Fig. 2. The four-membered ring system adopts a puckered conformation. The structure consists of hydrogen bonded layers of molecules in the *ab* plane (Fig. 3, Fig. 4). Each molecule is a hydrgen-bond donor and acceptor for three hydrogen bonds. Only classical hydrogen bonding was considered.

### Experimental

The title compound was recrystallized from methanol: [α]_D_^25^ -103.0 (*c* 0.23 in MeOH); m.p. 452–453 K.

### Refinement

In the absence of significant anomalous scattering, Friedel pairs were merged and the absolute configuration was assigned by the use of *D*-glucose as the starting material.

The H atoms were all located in a difference map, but those attached to C atoms were repositioned geometrically. The H atoms were initially refined with soft restraints on the bond lengths and angles to regularize their geometry (C—H in the range 0.93–0.98, N—H in the range 0.86–0.89 N—H to 0.86 O—H = 0.82 Å) and *U*_iso_(H) (in the range 1.2–1.5 times *U*_eq_ of the parent atom), after which the positions were refined with riding constraints.

### Crystal data

**Table:**

C_12_H_17_NO_3_	*F*(000) = 480
*M**_r_* = 223.27	*D*_x_ = 1.272 Mg m^−^^3^
Orthorhombic, *P*2_1_2_1_2_1_	Mo *K*α radiation, λ = 0.71073 Å
Hall symbol: P 2ac 2ab	Cell parameters from 1467 reflections
*a* = 6.2309 (2) Å	θ = 5–27°
*b* = 9.3918 (4) Å	µ = 0.09 mm^−^^1^
*c* = 19.9175 (9) Å	*T* = 150 K
*V* = 1165.56 (8) Å^3^	Prism, colourless
*Z* = 4	0.20 × 0.10 × 0.07 mm

### Data collection

**Table:**

Nonius KappaCCD diffractometer	1098 reflections with *I* > 2σ(*I*)
graphite	*R*_int_ = 0.065
ω scans	θ_max_ = 27.5°, θ_min_ = 5.2°
Absorption correction: multi-scan (*DENZO*/*SCALEPACK*; Otwinowski & Minor, 1997)	*h* = −8→8
*T*_min_ = 0.97, *T*_max_ = 0.99	*k* = −12→12
6149 measured reflections	*l* = −25→25
1541 independent reflections	

### Refinement

**Table:**

Refinement on *F*^2^	Hydrogen site location: inferred from neighbouring sites
Least-squares matrix: full	H-atom parameters constrained
*R*[*F*^2^ > 2σ(*F*^2^)] = 0.045	Method = modified Sheldrick, *w* = 1/[σ^2^(*F*^2^) + (0.05*P*)^2^ + 0.13*P*], where *P* = [max(*F*_o_^2^,0) + 2*F*_c_^2^]/3
*wR*(*F*^2^) = 0.102	(Δ/σ)_max_ = 0.0002627
*S* = 0.95	Δρ_max_ = 0.49 e Å^−^^3^
1541 reflections	Δρ_min_ = −0.49 e Å^−^^3^
146 parameters	Extinction correction: Larson (1970), Equation 22
0 restraints	Extinction coefficient: 400 (70)
Primary atom site location: structure-invariant direct methods	

### Special details

**Table:**

Experimental. The crystal was placed in the cold stream of an Oxford Cryosystems open-flow nitrogen cryostat (Cosier & Glazer, 1986) with a nominal stability of 0.1 K.

### Fractional atomic coordinates and isotropic or equivalent isotropic displacement parameters (Å^2^)

**Table:**

	*x*	*y*	*z*	*U*_iso_*/*U*_eq_	
O1	0.7910 (3)	0.89266 (18)	0.79718 (9)	0.0257	
C2	0.8327 (4)	0.7498 (3)	0.81847 (12)	0.0246	
C3	0.9587 (4)	0.6675 (3)	0.76640 (12)	0.0212	
O4	1.1771 (3)	0.71531 (19)	0.75988 (9)	0.0261	
C5	0.8472 (4)	0.6736 (3)	0.69895 (12)	0.0206	
N6	0.9436 (3)	0.5853 (2)	0.64366 (9)	0.0198	
C7	1.0678 (4)	0.6647 (3)	0.59325 (12)	0.0248	
C8	1.1061 (4)	0.5768 (3)	0.53082 (12)	0.0252	
C9	1.2467 (4)	0.4615 (3)	0.53077 (13)	0.0286	
C10	1.2791 (5)	0.3829 (3)	0.47302 (15)	0.0384	
C11	1.1727 (5)	0.4177 (3)	0.41474 (14)	0.0366	
C12	1.0312 (5)	0.5309 (3)	0.41392 (14)	0.0373	
C13	0.9984 (5)	0.6088 (3)	0.47187 (13)	0.0313	
C14	0.7214 (4)	0.5438 (3)	0.62186 (13)	0.0277	
C15	0.6349 (4)	0.5921 (3)	0.69017 (12)	0.0245	
O16	0.6117 (3)	0.48138 (19)	0.73784 (9)	0.0277	
H22	0.9101	0.7535	0.8624	0.0291*	
H21	0.6964	0.7000	0.8265	0.0280*	
H31	0.9716	0.5645	0.7806	0.0250*	
H51	0.8349	0.7719	0.6843	0.0251*	
H72	1.2057	0.6871	0.6139	0.0252*	
H71	0.9916	0.7537	0.5814	0.0268*	
H91	1.3207	0.4363	0.5701	0.0319*	
H101	1.3719	0.3075	0.4739	0.0480*	
H111	1.1995	0.3646	0.3761	0.0436*	
H121	0.9557	0.5547	0.3747	0.0440*	
H131	0.9009	0.6860	0.4710	0.0365*	
H141	0.7104	0.4397	0.6145	0.0348*	
H142	0.6670	0.5939	0.5830	0.0324*	
H151	0.5114	0.6524	0.6881	0.0289*	
H41	1.1628	0.8040	0.7623	0.0399*	
H161	0.4864	0.4499	0.7314	0.0409*	
H11	0.8789	0.9468	0.8187	0.0398*	

### Atomic displacement parameters (Å^2^)

**Table:**

	*U*^11^	*U*^22^	*U*^33^	*U*^12^	*U*^13^	*U*^23^
O1	0.0245 (9)	0.0213 (9)	0.0312 (9)	0.0011 (8)	−0.0042 (8)	−0.0039 (9)
C2	0.0288 (15)	0.0219 (14)	0.0231 (13)	−0.0006 (12)	−0.0021 (11)	0.0021 (11)
C3	0.0174 (12)	0.0210 (13)	0.0251 (13)	−0.0022 (11)	−0.0003 (11)	0.0004 (12)
O4	0.0193 (9)	0.0254 (10)	0.0336 (10)	0.0013 (8)	−0.0012 (8)	−0.0025 (9)
C5	0.0242 (13)	0.0189 (12)	0.0187 (11)	−0.0011 (11)	0.0015 (11)	−0.0004 (11)
N6	0.0205 (10)	0.0233 (11)	0.0157 (10)	−0.0008 (10)	−0.0005 (9)	−0.0030 (10)
C7	0.0226 (13)	0.0283 (15)	0.0236 (13)	−0.0042 (13)	0.0003 (12)	−0.0009 (12)
C8	0.0246 (14)	0.0304 (15)	0.0206 (12)	−0.0055 (13)	0.0004 (11)	0.0012 (12)
C9	0.0224 (13)	0.0402 (17)	0.0232 (13)	0.0039 (13)	0.0016 (12)	0.0029 (13)
C10	0.0325 (16)	0.0423 (18)	0.0404 (16)	0.0075 (15)	0.0068 (15)	−0.0048 (16)
C11	0.0372 (16)	0.0458 (19)	0.0267 (14)	0.0000 (16)	0.0051 (14)	−0.0056 (15)
C12	0.0399 (17)	0.0489 (19)	0.0232 (14)	−0.0010 (17)	−0.0029 (14)	−0.0025 (14)
C13	0.0350 (16)	0.0333 (16)	0.0256 (13)	0.0041 (14)	−0.0033 (12)	0.0017 (14)
C14	0.0212 (13)	0.0347 (16)	0.0273 (14)	−0.0005 (13)	−0.0055 (12)	−0.0009 (13)
C15	0.0174 (12)	0.0286 (15)	0.0275 (12)	0.0059 (12)	−0.0002 (11)	0.0017 (13)
O16	0.0215 (9)	0.0282 (10)	0.0335 (10)	−0.0042 (8)	−0.0010 (9)	0.0070 (9)

### Geometric parameters (Å, °)

**Table:**

O1—C2	1.431 (3)	C8—C9	1.393 (4)
O1—H11	0.862	C8—C13	1.385 (4)
C2—C3	1.513 (3)	C9—C10	1.382 (4)
C2—H22	1.000	C9—H91	0.939
C2—H21	0.982	C10—C11	1.376 (4)
C3—O4	1.439 (3)	C10—H101	0.914
C3—C5	1.514 (3)	C11—C12	1.381 (4)
C3—H31	1.011	C11—H111	0.932
O4—H41	0.839	C12—C13	1.382 (4)
C5—N6	1.504 (3)	C12—H121	0.939
C5—C15	1.538 (3)	C13—H131	0.945
C5—H51	0.971	C14—C15	1.532 (3)
N6—C7	1.471 (3)	C14—H141	0.991
N6—C14	1.503 (3)	C14—H142	0.967
C7—C8	1.512 (3)	C15—O16	1.416 (3)
C7—H72	0.976	C15—H151	0.956
C7—H71	0.990	O16—H161	0.844
			
C2—O1—H11	106.9	C7—C8—C13	120.2 (2)
O1—C2—C3	111.7 (2)	C9—C8—C13	118.2 (2)
O1—C2—H22	108.3	C8—C9—C10	120.5 (3)
C3—C2—H22	111.6	C8—C9—H91	120.3
O1—C2—H21	109.7	C10—C9—H91	119.2
C3—C2—H21	108.5	C9—C10—C11	120.3 (3)
H22—C2—H21	106.9	C9—C10—H101	119.4
C2—C3—O4	113.2 (2)	C11—C10—H101	120.3
C2—C3—C5	110.5 (2)	C10—C11—C12	120.0 (3)
O4—C3—C5	110.0 (2)	C10—C11—H111	118.9
C2—C3—H31	109.8	C12—C11—H111	121.1
O4—C3—H31	104.4	C11—C12—C13	119.5 (3)
C5—C3—H31	108.7	C11—C12—H121	120.9
C3—O4—H41	101.8	C13—C12—H121	119.6
C3—C5—N6	116.5 (2)	C8—C13—C12	121.4 (3)
C3—C5—C15	118.5 (2)	C8—C13—H131	119.5
N6—C5—C15	89.19 (17)	C12—C13—H131	119.1
C3—C5—H51	109.8	N6—C14—C15	89.48 (18)
N6—C5—H51	109.6	N6—C14—H141	111.2
C15—C5—H51	111.8	C15—C14—H141	113.5
C5—N6—C7	115.48 (19)	N6—C14—H142	115.3
C5—N6—C14	89.22 (17)	C15—C14—H142	116.3
C7—N6—C14	114.8 (2)	H141—C14—H142	109.7
N6—C7—C8	111.6 (2)	C5—C15—C14	86.89 (19)
N6—C7—H72	106.5	C5—C15—O16	112.1 (2)
C8—C7—H72	109.0	C14—C15—O16	114.5 (2)
N6—C7—H71	109.8	C5—C15—H151	113.7
C8—C7—H71	109.9	C14—C15—H151	114.9
H72—C7—H71	109.9	O16—C15—H151	112.5
C7—C8—C9	121.6 (2)	C15—O16—H161	104.4

### Hydrogen-bond geometry (Å, °)

**Table:**

*D*—H···*A*	*D*—H	H···*A*	*D*···*A*	*D*—H···*A*
C11—H111···O4^i^	0.93	2.55	3.457 (4)	164
C15—H151···O4^ii^	0.96	2.59	3.377 (4)	139
O4—H41···O16^iii^	0.84	2.18	2.825 (4)	134
O16—H161···O1^iv^	0.84	1.90	2.735 (4)	171
O1—H11···N6^iii^	0.86	1.86	2.719 (4)	171

Symmetry codes: (i) −*x*+5/2, −*y*+1, *z*−1/2; (ii) *x*−1, *y*, *z*; (iii) −*x*+2, *y*+1/2, −*z*+3/2; (iv) −*x*+1, *y*−1/2, −*z*+3/2.
